# Unmanipulated haploidentical in comparison with matched unrelated donor stem cell transplantation in patients 60 years and older with acute myeloid leukemia: a comparative study on behalf of the ALWP of the EBMT

**DOI:** 10.1186/s13045-018-0598-0

**Published:** 2018-04-16

**Authors:** Nicole Santoro, Myriam Labopin, Federica Giannotti, Gerard Ehninger, Dietger Niederwieser, Arne Brecht, Matthias Stelljes, Nicolaus Kröger, Herman Einsele, Matthias Eder, Michael Hallek, Bertram Glass, Jürgen Finke, Fabio Ciceri, Mohamad Mohty, Annalisa Ruggeri, Arnon Nagler

**Affiliations:** 1 Department of Hematology and Cell Therapy, Saint-Antoine Hospital, Paris, France; 2 Section of Hematology, Department of Medicine, University of Perugia, Centro Ricerche Emato-Oncologiche (CREO), Perugia, Italy; 3 ALWP office, Hôpital Saint-Antoine, Paris, France; 4 Medical Clinic and Policlinic I, University Hospital Carl Gustav Carus, Technical University Dresden, Dresden, Germany; 5 Department of Hematology and Oncology, University of Leipzig, Leipzig, Germany; 6 Center for Blood Stem Cell and Bone Marrow Transplant, DKD Helios Clinic Wiesbaden, Wiesbaden, Germany; 7 Department of Medicine A/Hematology and Oncology, University of Muenster, Muenster, Germany; 8 Department of Stem Cell Transplantation, University Medical Center Hamburg-Eppendorf, Hamburg, Germany; 9 Department of Internal Medicine II, University Hospital Wurzburg, Würzburg, Germany; 10 Department of Hematology, Hemostasis, Oncology and Stem Cell Transplantation, Hannover Medical School, Hannover, Germany; 11 Department I of Internal Medicine and Center of Integrated Oncology Cologne-Bonn, German CLL Study Group, University Hospital of Cologne, Cologne, Germany; 12 Department of Hematology and Oncology, Asklepios Klinik St. Georg Hamburg, Hamburg, Germany; 13 Department of Medicine-Hematology, Oncology, University of Freiburg, Freiburg, Germany; 14 Hematology and Bone Marrow Transplantation Unit, IRCCS San Raffaele Scientific Institute, Milan, Italy; 15 Department of Pediatric Hematology and Oncology, IRCCS Bambino Gesù Children’s Hospital, Piazza S Onofrio, 4, 00165 Rome, Italy; 16 Department of Hematology and Bone Marrow Transplantation, Chaim Sheba Medical Center, Tel Hashomer, Israel

**Keywords:** MUD, Haploidentical, Allogeneic stem cell transplantation, Acute myeloid leukemia, Elderly

## Abstract

**Background:**

Acute myeloid leukemia (AML) is both more common and with more biologically aggressive phenotype in the elderly. Allogenic stem cell transplantation (allo-SCT) is the best treatment option in fit patients. Either HLA-matched unrelated donor (MUD) or haploidentical (Haplo) donor are possible alternative for patients in need.

**Methods:**

We retrospectively compared non-T-cell-depleted Haplo (*n* = 250) to 10/10 MUD (*n* = 2589) in AML patients ≥ 60 years.

**Results:**

Median follow-up was 23 months. Disease status at transplant differs significantly between the two groups (*p* < 10^−4^). Reduced intensity conditioning (RIC) was administrated to 73 and 77% of Haplo and MUD, respectively (*p* = 0.23). Stem cell source was the bone marrow (BM) in 52% of the Haplo and 6% of MUD (*p* < 10^−4^). Anti-thymocyte globulin (ATG) was most frequently used in MUD (*p* < 10^−4^) while post-Tx cyclophosphamide (PT-Cy) was given in 62% of Haplo. Engraftment was achieved in 90% of the Haplo vs 97% of MUD (*p* < 10^−4^). In multivariate analysis, no significant difference was found between Haplo and MUD for acute (a)graft versus host disease (GVHD) grade II–IV, relapse incidence (RI), non-relapse mortality (NRM), leukemia free survival (LFS), graft-versus-host-free-relapse free survival (GRFS), and overall survival (OS). Extensive chronic (c)GVHD was significantly higher for MUD as compared to Haplo (HR 2, *p* = 0.01, 95% CI 1.17–3.47). A propensity score analysis confirmed the higher risk of extensive cGVHD for MUD without differences for other outcomes.

**Conclusions:**

Allo-SCT from both Haplo and MUD are valid option for AML patients ≥ 60 years of age with similar results. Transplantation from MUD was associated with higher extensive cGVHD. Our findings suggest that Haplo is a suitable and attractive graft source for patients≥ 60 with AML in need of allo-SCT.

## Background

Acute myeloid leukemia (AML) is primarily a disease of the elderly, with a median age at diagnosis of 68–72 years [1]. For these patients, prognosis still remains very poor. Recent data indicate a median survival of 3 months for patients > 65 years, with slightly higher estimates for patients aged 66–75 years (about ~ 6 months) than in patients 76–89 year olds (in the neighborhood of 2.5 months) and merely 5% of the patients are alive 5 years from diagnosis [2].

In elderly, AML is associated with poorer prognosis than in the younger, due to the frequent adverse genetic or epigenetic features present at diagnosis and the increased non-relapse mortality (NRM) [3]. Although most elderly AML patients still succumb to their disease, improvements in the prognosis have been documented in recent years, due to better supportive care, management of infections, and patient selection for chemotherapy based on comorbidity scores [4]. Allogenic stem cell transplantation (allo-SCT) remains the most effective anti-leukemic treatment in AML, but the toxicity of the procedure and the difficulty to allocate a suitable donor have limited allo-SCT to a small fraction of patients in need [5]. Both the development of reduced conditioning regimens (RIC) [6–8] and the use of alternative donors improved the accessibility to transplant for high-risk patients in need. Indeed, a recent study reports that over the past decade, utilization and survival after allo-SCT have significantly increased in patients ≥ 70 years [9].

Availability of a donor for this high-risk patient population is still a challenge. HLA matched related donor (MRD) transplants remain the best choice for optimal transplant outcome, but only approximately 25–30% of patients have such a donor [10]. Moreover, in the elderly patients, availability of MRD is even lower, due to the increased age of family members as well as comorbidities and contraindications for stem cell donation. Other donor options include HLA-matched unrelated donors (MUD), HLA-mismatched unrelated donors 9/10 (MMUD), umbilical cord blood units (UCB), and haploidentical family donors. MUD or MMUD are frequently used when a suitable MRD is lacking, with similar transplantation outcomes [11–14]. However, these reports focus mainly on young patients receiving myeloablative conditioning (MAC), and there are only few data from older populations [15]. In the absence of MRD, MUD is one of the possible choices but the probability to find a donor is estimated to be around 75% in Caucasians, 46% in Middle East or North African origin, and only 16% in African-Americans [16]. In addition, the search may take few weeks or even months while waiting for a volunteer donor; the leukemia may reoccur.

Over the last recent years, haploidentical donors have been increasingly used as a valid alternative for allo-SCT [17]. Unmanipulated haploidentical transplant (Haplo-SCT) without T-cell depletion (TCD) is currently used more frequently either with anti-thymocyte globulins (ATG) or post-transplant cyclophosphamide (PT-Cy) as graft versus host disease (GVHD) prophylaxis [18–20]. The optimization of conditioning regimens has further extended the use of Haplo-SCT to older patients and those with significant pre-transplant comorbidities. However, while the feasibility of allo-SCT from an MRD using reduced intensity conditioning (RIC) regimens in elderly patients was demonstrated in several studies [21–23], only few studies reported the outcomes of Haplo-SCT in elderly patients mainly using RIC and PT-Cy-based GVHD prophylaxis [24–29].

With the aim to better define the role of alternative donors in elderly patients with AML, we performed a large registry-based study, with the European Society of Bone Marrow Transplantation (EBMT)-Acute Leukemia Working Party (ALWP), comparing non-TCD Haplo-SCT to transplants from MUD for AML patients 60 years and older.

## Methods

### Study design and definition

This is a retrospective registry-based analysis on behalf of the ALWP of EBMT. The EBMT is a non-profit, scientific society representing more than 600 transplant centers mainly in Europe that are required to report all consecutive stem cell transplantations and follow-ups once a year. Data are entered, managed, and maintained in a central database with internet access; each EBMT center is represented in this database. Audits are routinely performed to determine the accuracy of the data. Patients provide informed consent authorizing the use of their personal information for research purposes.

Eligibility criteria included all adults ≥ 60 years with AML, who underwent a first allo-SCT between January 2007 and December 2014, using either a 10/10 MUD or Haplo from a family donor (recipient-donor number of mismatches ≥ 2). Transplants were performed in 210 EBMT centers.

MAC was defined as a regimen containing either total body irradiation (TBI) with a dose greater than 6 Gy, a total dose of oral busulfan (Bu) greater than 8 mg/kg, or a total dose of intravenous Bu greater than 6.4 mg/kg or melphalan at doses > 140 mg/m^2^. In addition, regimens containing two alkylating agents were considered as MAC. All other regimens were defined as RIC [30]. Cytogenetics abnormalities were classified according to the 2010 European Leukemia Net cytogenetic classification system [31].

### Endpoints

The primary endpoint was leukemia-free survival (LFS). Secondary endpoints were overall survival (OS), refined graft-versus-host-free, relapse-free survival (GRFS), neutrophil engraftment, acute (a)GVHD and chronic (c)GVHD, relapse incidence (RI), and non-relapse mortality (NRM). LFS was defined as the interval from Haplo-SCT to either relapse or death in remission. OS was defined as the time to death from all causes. GRFS events have been defined as grade III–IV aGVHD, severe cGVHD, disease relapse, or death from any cause after SCT [32]. Engraftment was defined as the first of three consecutive days with an absolute neutrophil count > 0.5 × 10^9^/l. aGVHD was graded according to the modified Glucksberg criteria [33] and cGVHD according to the revised Seattle criteria [34].

### Statistical analysis

Patient-, disease-, and transplant-related variables were compared between the two groups (Haplo or MUD) using the chi-square statistic for categorical variables and the Mann-Whitney test for continuous variables. Factors that differ significantly between the two groups with *p* values of < 0.05 and all factors known as potential prognostic factors were included in the final models. Cumulative incidence (CI) of relapse and NRM was calculated from the date of transplant to the date of relapse or death in remission, respectively, with the other event being the competing risk. For studying GVHD, both relapse and death were considered as competing events. Univariate comparisons of time-dependent endpoints were done using the log-rank test for OS and LFS and GRFS and the Gray’s test for cumulative incidence functions. A multivariate analysis was performed using Cox proportional hazards model. Variables were included in the multivariate model if they were conceptually important or if they approached or attained statistical significance by univariate analysis. All tests are two-sided. The type I error rate was fixed at 0.05 for determination of factors associated with time to event. In order to test for a center effect, we introduced a random effect or frailty for each center into the model. Statistical analyses were performed with the SPSS 22 (SPSS Inc./IBM, Armonk, NY, USA) and R 3.2.3 (R Development Core Team, Vienna, Austria) software package.

To allow for potential confounding factors between treatments that could influence outcome, propensity score matching was also performed [35]. The following factors were included in the propensity score model: patient age, year of transplant, cytogenetics, status at transplant (Tx), sex, female donor to male recipient vs other combinations, Karnofsky performance status less or more than 90%, patient and donor CMV serology, stem cell source (peripheral blood stem cell (PBSC) vs BM), use of in vivo T-cell depletion, conditioning (MAC vs RIC), and previous autograft. Owing to the significant differences in baseline characteristics between the haplo and MUD groups, caliper matching was fixed to 0.2. The purpose of the propensity score matching strategy was to reduce confounding effects of these variables and strengthen causal inferences. Propensity score analysis was performed using the “MatchIt” (https://cran.r-project.org/web/packages/MatchIt/index.html). Comparisons between the two match-paired groups were stratified on matching group.

## Results

### Patients, disease, and transplant characteristics

We analyzed 250 AML patients ≥ 60 years, receiving Haplo-SCT and 2589 patients transplanted from MUD between 2007 and 2014. Patients and transplant characteristics are summarized in Table 1.

**Table 1:** Patients and donors characteristics

	Haplo	MUD 10/10	*p* value
Number	250	2589	
Follow-up	23.18 (1.8–93.9)	23.02 (0.2–128)	
Year of Tx	2013 (2006–2014)	2012(2001–2014)	< 10^− 4^
Time to Tx (months)	9 (5.1–18.7)	6.8 (4.6–15)	0.001
Patient sex			
Male	156 (62%)	1471 (57%)	0.093
Female	94 (38%)	1114 (43%)	
Missing	0	4	
Performance status			
KPS ≤ 90	97 (41%)	723 (28%)	0.001
KPS ≥ 90	139 (59%)	1670 (65%)	
Missing	14 (0%)	196 (7%)	
Age	65(62.3–66.9)	64.8(62.2–67.6)	0.756
Disease status			
CR1	95 (38%)	1377 (53%)	< 10^−4^
CR ≥ 2	46 (18%)	436 (17%)	
Active disease	109 (44%)	776 (30%)	
Missing	0	0	
Cytogenetics			
Good	17 (10%)	77 (5%)	0.036
Intermediate	28 (17%)	210 (15%)	
Poor	31 (18%)	221 (16%)	
Secondary AML	92 (55%)	902 (64%)	0.535
Missing	82	1179	
Previous autologus Tx	21 (8%)	49 (2%)	< 10^−4^
Sex mismatch D/R			
No F->M	197 (79%)	2210 (88%)	< 10^− 4^
F->M	53 (21%)	297 (12%)	
Missing	0	82	

*Abbreviations*: *Haplo* haploidentical; *MUD*, matched unrelated donor; *TX*, transplant; *KPS*, Karnofsky performance status; *CR*, complete remission; *AML*, acute myeloid leukemia; *F*, female; *M*, male; *D*, donor; *R*, recipient

Median follow-up was 23 months for both Haplo and MUD. Haplo-SCT were performed more recently, median year of transplant 2013 vs 2012 for MUD, respectively (*p* < 10^−4^). Median time from diagnosis to transplant was longer for Haplo (9 vs 6.8 months, respectively, *p* = 0.001). The majority of patients had a Karnofsky performance status (KPS) ≥ 90%, being 59% for Haplo and 70% for MUD, *p* = 0.001. The median age was 65 years (range 60–78) for both groups. Disease status was significantly different between the two groups (*p* < 10^−4^). Secondary AML was diagnosed in 37% of haplo and 35% of MUD. As for cytogenetics, it was intermediate risk in 17% of Haplo and 15% of MUD, while 18 and 16% of the Haplo and MUD harbor poor-risk cytogenetics, respectively (*p* = 0.03).

Stem cell source, GVHD prophylaxis, and conditioning regimens are provided in Table 2.

**Table 2:** Stem cell source, GVHD prophylaxis, and conditioning regimens

	Haplo, *n* (%)	MUD 10/10, *n* (%)	*p* value
Stem cell source			
BM	129 (52)	167 (6)	< 10^−4^
PBSC	121 (48)	2422 (94)	
Missing	0	0	
GVHD prophylaxis			
Csa based	4 (2)	426 (17)	< 10^−4^
Csa + Mtx	9 (4)	656 (26)	
Csa + Mmf ± others	12 (5)	1123 (44)	
CSA + MMF ± MTX	16 (7)	29 (1)	
Tacro alone	1 (0)	20 (1)	
Mmf + Tacro/Mmf + Siro	38 (16)	185 (7)	
Mtx + Tacro	0 (0)	44 (2)	
Pt-Cy based	156 (65)	22 (1)	
Others	14 (1)	84 (1)	
In vivo TCD			
No	181 (74)	648 (25)	< 10^−4^
Yes	63 (26)	1915 (75)	
Missing	6	26	
Conditioning regimens			
MAC	66 (27)	591 (23)	0.238
Bu-Cy/BuFlu	7	253	
TBF	31	8	
Flu-Mel	7	81	
TBI based	4	61	
Other	17	188	
RIC	182 (73)	1948 (77)	
Bu-Flu	10	635	
TBF	55	19	
Flu-Mel	9	414	
TBI based	55	457	
Other	53	423	

*Abbrevations*: *Haplo*, haploidentical; *MUD*, matched unrelated donor; *BM*, bone marrow; *PBSC*, peripheral blood stem cell; *GVHD*, graft versus host disease; *CSA*, cyclosporine; *MTX*, methotrexate; *MMF*, mycophenolate; *TACRO*, tacrolimus; *SIRO*, sirolimus; *PTCY*, post-transplant cyclophosphamide; *TCD*, T-cell depletion; *MAC*, myeloablative conditioning; *BU*, busulphan, *CY*, cyclophosphamide; *FLU*, fludarabine; *TBF*, thiotepa busulphan fludarabine; *MEL*, melphalan; *TREO*, treosulphan; *FLAMSA*, fludarabine, amsacrine, and cytarabine; *TBI*, total body irradiation; *RIC*, reduced intensity conditioning

Stem cell source was the bone marrow (BM) in 52% of Haplo and 6% of MUD. Most of the MUD (94%) received PBSC grafts (p < 10^−4^). Cyclosporine and mycophenolate were used as the main GVHD prophylaxis in the MUD group (44.5%) while PT-CY for GVHD prophylaxis was used in 65% of Haplo. Finally, T-cell depletion with ATG significantly differs between the two groups (74 vs 26%, respectively, *p* < 10^−4^) (Table 2). In order to overcome these differences, the propensity score technique was applied and we were able to match 225 haplo with 450 MUD using propensity score matching.

### Engraftment and acute and chronic graft versus host disease

The 60 days CI of neutrophil engraftment was 90% for the Haplo and 97% for the MUD (*p* < 10^−4^).

In the multivariate analysis (Table 3), the risk of grade II–IV aGVHD (HR 1.17, *p* = 0.37, 95% CI 0.82–1.65) and cGVHD (HR1.21, *p* = 0.28, 95% CI 0.84–1.75) was not associated to the type of donor. However, recipients of MUD experienced higher risk of extensive cGVHD compared to Haplo (HR 2, *p* = 0.01, 95% CI 1.17–3.47).

**Table 3:** Multivariate analysis

	HR	CI	*p*
LFS			
MUD vs Haplo	0.94	0.76–1.17	0.630
Year of Tx	1.01	0.98–1.03	0.445
Age (per 10 years)	1.10	0.95–1.27	0.173
Status at Tx			
CR ≥ 2 vs CR1	1.22	1.05–1.42	0.009
Advanced vs CR1	1.67	1.48–1.89	< 10^− 4^
Cytogenetics			
Intermediate vs good	1.10	0.76–1.60	0.590
Poor vs good	1.79	1.25–2.56	0.001
Secondary vs good	1.49	1.06–2.08	0.019
Missing vs good	1.40	1.01–1.95	0.043
Female D/male R	1.09	0.93–1.27	0.247
KPS ≥ 90%	0.85	0.76–0.96	0.009
PBSC vs BM	1.04	0.86–1.25	0.670
In vivo TCD	0.93	0.82–1.05	0.288
Previous autograft	1.36	1.00–1.86	0.045
RIC vs MAC	1.04	0.92–1.18	0.468
Center (frailty)			0.292
OS			
MUD vs Haplo	0.87	0.68–1.10	0.244
Year of Tx	1.01	0.98–1.04	0.392
Age (per 10 years)	1.16	1.00–1.34	0.046
Status at Tx			
CR ≥ 2 vs CR1	1.16	0.99–1.37	0.059
Advanced vs CR1	1.62	1.42–1.85	< 10^−4^
Cytogenetics			
Intermediate vs good	1.05	0.71–1.54	0.792
Poor vs good	1.66	1.14–2.40	0.007
Secondary vs good	1.40	0.99–1.98	0.057
Missing vs good	1.32	0.94–1.87	0.105
Female D/male R	1.14	0.97–1.34	0.093
KPS ≥ 90%	0.83	0.74–0.94	0.003
PBSC vs BM	1.01	0.83–1.24	0.893
in vivo TCD	0.93	0.81–1.08	0.372
Previous autograft	1.39	1.00–1.93	0.043
RIC vs MAC	1.12	0.97–1.28	0.096
Center (frailty)			0.062
RI			
MUD vs Haplo	1.06	0.76–1.47	0.699
Year of Tx	0.99	0.96–1.03	0.790
Age (per 10 years)	0.87	0.71–1.08	0.230
Status at Tx			
CR ≥ 2 vs CR1	1.26	1.01–1.57	0.033
Advanced vs CR1	1.96	1.65–2.34	< 10^−4^
Cytogenetics			
Intermediate vs good	0.95	0.57–1.60	0.869
Poor vs good	2.11	1.29–3.43	0.002
Secondary vs good	1.35	0.85–2.14	0.202
Missing vs good	1.31	0.83–2.06	0.241
Female D/male R	0.88	0.69–1.11	0.285
KPS ≥ 90%	0.96	0.82–1.14	0.711
PBSC vs BM	1.02	0.77–1.34	0.865
in vivo TCD	1.04	0.86–1.25	0.662
Previous autograft	1.16	0.71–1.88	0.546
RIC vs MAC	0.91	0.76–1.10	0.374
Center (frailty)			0.135
NRM			
MUD vs Haplo	0.75	0.54–1.05	0.095
Year of Tx	1.02	0.98–1.06	0.243
Age (per 10 years)	1.34	1.10–1.63	0.003
Status at Tx			
CR ≥ 2 vs CR1	1.18	0.94–1.47	0.135
Advanced vs CR1	1.45	1.21–1.74	< 10^−4^
Cytogenetics			
Intermediate vs good	1.29	0.75–2.22	0.353
Poor vs good	1.46	0.84–2.51	0.172
Secondary vs good	1.63	0.99–2.68	0.051
Missing vs good	1.49	0.91–2.43	0.111
Female D/male R	1.34	1.08–1.65	0.005
KPS ≥ 90%	0.75	0.63–0.89	0.001
PBSC vs BM	1.08	0.82–1.43	0.564
In vivo TCD	0.85	0.70–1.04	0.122
Previous autograft	1.62	1.07–2.46	0.022
RIC vs MAC	1.19	0.98–1.43	0.066
Center (frailty)			0.015
GRFS			
MUD vs Haplo	1.18	0.95–1.47	0.125
Year of Tx	0.99	0.97–1.02	0.792
Age (per 10 years)	1.03	0.90–1.18	0.606
Status at Tx			
CR ≥ 2 vs CR1	1.16	1.00–1.34	0.038
Advanced vs CR1	1.64	1.45–1.84	< 10^−4^
Cytogenetics			
Intermediate vs good	1.24	0.87–1.75	0.223
Poor vs good	1.69	1.20–2.38	0.002
Secondary vs good	1.52	1.11–2.09	0.008
Missing vs good	1.45	1.06–1.98	0.019
Female D/male R	1.05	0.90–1.21	0.516
KPS ≥ 90%	0.85	0.76–0.94	0.003
PBSC vs BM	1.09	0.91–1.32	0.313
In vivo TCD	0.75	0.66–0.85	< 10^−4^
Previous autograft	1.32	0.97–1.80	0.072
RIC vs MAC	1.00	0.88–1.13	0.933
Center (frailty)			0.093
aGVHD II–IV			
MUD vs Haplo	1.17	0.82–1.65	0.374
Year of Tx	1.00	0.96–1.04	0.845
Age (per 10 years)	0.89	0.71–1.11	0.326
Status at Tx			
CR ≥ 2 vs CR1	1.00	0.79–1.27	0.942
advanced vs CR1	1.15	0.95–1.40	0.135
Cytogenetics			
Intermediate vs good	1.34	0.77–2.34	0.296
Poor vs good	1.45	0.83–2.53	0.180
Secondary vs good	1.40	0.84–2.34	0.192
Missing vs good	1.30	0.78–2.16	0.303
Female D/male R	1.10	0.88–1.39	0.378
KPS ≥ 90%	0.87	0.73–1.05	0.152
PBSC vs BM	1.01	0.75–1.35	0.954
In vivo TCD	0.70	0.57–0.85	0.001
Previous autograft	2.18	1.42–3.36	< 10^−4^
RIC vs MAC	1.02	0.82–1.25	0.853
Center (frailty)			0.011
aGVHD III–IV			
MUD vs Haplo	1.52	0.85–2.71	0.154
Year of Tx	0.97	0.91–1.03	0.369
Age (per 10 years)	0.98	0.68–1.41	0.935
Status at Tx			
CR ≥ 2 vs CR1	1.23	0.83–1.84	0.290
Advanced vs CR1	1.78	1.31–2.43	< 10^−4^
Cytogenetics			
Intermediate vs good	2.27	0.77–6.68	0.133
Poor vs good	1.67	0.55–5.04	0.361
Secondary vs good	2.03	0.73–5.67	0.173
Missing vs good	1.69	0.61–4.70	0.311
Female D/male R	1.02	0.69–1.51	0.895
KPS ≥ 90%	0.68	0.52–0.91	0.009
PBSC vs BM	1.20	0.71–2.02	0.478
In vivo TCD	0.63	0.46–0.86	0.004
Previous autograft	2.07	1.03–4.15	0.039
RIC vs MAC	0.76	0.53–1.09	0.147
Center (frailty)			0.054
cGVHD			
MUD vs Haplo	1.22	0.84–1.75	0.281
Year of Tx	0.97	0.94–1.01	0.228
Age (per 10 years)	0.99	0.80–1.23	0.964
Status at Tx			
CR ≥ 2 vs CR1	1.05	0.84–1.30	0.669
Advanced vs CR1	1.12	0.92–1.37	0.251
Cytogenetics			
Intermediate vs good	1.72	1.01–2.91	0.042
Poor vs good	1.74	1.01–2.99	0.045
Secondary vs good	1.88	1.14–3.10	0.013
Missing vs good	1.66	1.01–2.72	0.043
Female D/male R	1.02	0.81–1.28	0.860
KPS ≥ 90%	1.09	0.91–1.31	0.310
PBSC vs BM	1.48	1.08–2.03	0.013
In vivo TCD	0.57	0.47–0.70	< 10^−4^
Previous autograft	1.64	0.99–2.70	0.050
RIC vs MAC	0.82	0.67–1.02	0.077
Center (frailty)			0.001
Extensive cGVHD			
MUD vs Haplo	2.02	1.17–3.47	0.011
Year of Tx	0.97	0.92–1.02	0.298
Age (per 10 years)	1.05	0.77–1.42	0.740
Status at Tx			
CR ≥ 2 vs CR1	0.92	0.67–1.26	0.618
Advanced vs CR1	0.97	0.73–1.30	0.874
Cytogenetics			
Intermediate vs good	1.15	0.56–2.37	0.693
Poor vs good	1.39	0.67–2.90	0.371
Secondary vs good	1.27	0.64–2.49	0.481
Missing vs good	1.11	0.57–2.16	0.751
Female D/male R	0.94	0.66–1.34	0.751
KPS ≥ 90%	1.00	0.77–1.29	0.997
PBSC vs BM	1.30	0.83–2.05	0.245
In vivo TCD	0.35	0.27–0.46	< 10^−4^
Previous autograft	1.19	0.54–2.60	0.658
RIC vs MAC	0.74	0.54–1.01	0.060
Center(frailty)			0.022

*Abbreviations*: *LFS*, leukemia-free survival; *OS*, overall survival; *RI*, relapse incidence; *NRM*, non-relapse mortality; *GRFS*, graft-versus-host-free-relapse free survival; *a*, acute; *c*, chronic; *GVHD*, graft versus host disease; *MUD*, matched unrelated donor; *Haplo*, haploidentical; *Tx*, transplant; *CR*, complete remission; *KPS*, Karnofsky performance status; *PBSC*, peripheral blood; *BM*, bone marrow; *D*, donor; *R*, recipient; *TCD*, T cell depletion; *RIC*, reduced intensity conditioning; *MAC*, myeloablative conditioning

In vivo T-cell depletion was associated with a reduced risk of aGVHD (HR 0.699, 95% CI 0.57–0.858, *p* < 0.01) and cGVHD (HR 0.57, 95% CI 0.4–0.7, *p* < 0.01) while the use of PBSC as stem cell source increased risk of cGVHD (HR 1.48, 95% CI 1.08–2.03, *p* = 0.01).

**Fig. 1:**
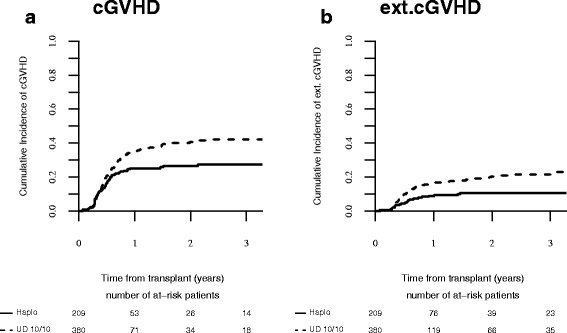
**a**) cGVHD; **b**) extensive cGVHD after MUD and Haplo SCT in AML patients ≥ 60 years after matched pair analysis

These findings were confirmed in the pair-matched analysis, with a significantly increased risk of extensive cGVHD (20.5 vs 10.7%, *p* = 0.0041) in MUD vs Haplo (Fig. 1).

### Relapse incidence and NRM

In multivariate analysis (Table 3), there was no difference in RI according to the type of donor. Factors independently associated with increased risk of relapse were disease status at transplantation (HR 1.96, 95% CI 1.65–2.34, *p* < 0.01) and cytogenetics risk (poor vs good) (*p* = 0.002) (Table 3).

The most common causes of death were disease recurrence (35% in the haplo and 45% in the MUD group, respectively), infections (35 vs 26%), and GVHD (18 vs 16%).

**Fig. 2:**
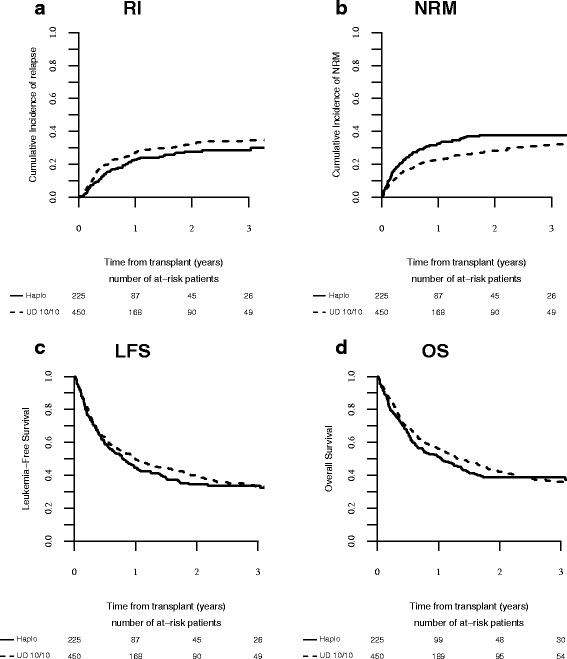
**a**) RI; **b**) NRM; **c**) LFS; **d**) OS after MUD and Haplo SCT in AML patients ≥ 60 years after matched pair analysis

In multivariate analysis, the type of donor did not influence the risk of NRM. Increased age (HR 1.342, *p* = 0.003, 95% CI 1.102–1.634), KPS (HR 0.753, 95% CI 0.637–0.891, *p* = 0.0009), and advanced disease status (HR 1.454, 95% CI 1.212–1.743, *p* < 10^−4^) (Table 3) were the factors independently associated with increase in mortality. These results were confirmed in the pair-matched analysis (Table 4, Fig. 2a, b).

**Table 4:** Propensity score analysis for LFS, OS, RI, NRM, GRFS, aGVHD II–IV, aGVHD III–IV, cGVHD, and ext. cGVHD

	MUD	Haplo	*p* value
LFS	39.9% [34.7–45.1]	34.6% [27.9–41.3]	0.67
OS	42% [36.7–47.4]	38.7% [31.8–45.5]	0.33
RI	31.9% [27.2–36.7]	27.7% [21.7–34]	0.17
NRM	28.2% [23.6–32.9]	37.7% [31.1–44.3]	0.06
GRFS	24.8% [20.3–29.4]	30.2% [23.7–36.6]	0.15
aGVHD II-IV	33.1% [28.6–37.6]	30.5% [24.4–36.8]	0.28
cGVHD	40.6% [35.3–45.9]	26.5% [20.5–32.7]	0.24
ext cGVHD	20.5% [16.2–25.1]	10.7% [6.8–15.5]	0.041

*Abbreviations*: *LFS*, leukemia free survival; *OS*, overall survival; *RI*, relapse incidence; *NRM*, non-relapse mortality; *GRFS*, graft-versus-host-free-relapse free survival; *a*, acute; *c*, chronic; *GVHD*, graft versus host disease; *ext.*, extensive

### OS, LFS, and GRFS

The median follow-up for survivors was 23 months. In adjusted multivariate analysis, OS (HR1.18, CI 0.686–1.102, *p* = 0.2481), LFS (HR 0.94, CI 0.767–1.174, *p* = 0.63), and GRFS (HR1.18, CI 0.954–1.47, *p* = 0.125) (Table 3) were comparable according to the type of donor. These results were confirmed in the pair-matched population (Table 4, Fig. 2c, d). Other independent factors influencing LFS, OS, and GRFS were disease status at transplantation (HR 1.67, 95% CI 1.48–1.89, *p* < 0.01; HR 1.62, 95% CI 1.42–1.85, *p* < 0.01; HR 1.64, 95% CI 1.45–1.84, *p* < 0.01), cytogenetic risk (HR 1.79, 95% CI 1.25–2.56, *p* < 0.01; HR 1.66, 95% CI 1.14–2.40, *p* < 0.01; HR 1.69, 95% CI 1.20–2.38, *p* < 0.01), KPS (HR 0.85, 95% CI 0.76–0.96, *p* = 0.009; HR 0.83, 95% CI 0.74–0.94, *p* = 0.003; HR 0.85, 95% CI 0.76–0.94, *p* = 0.003). Previous autograft was associated with lower OS (HR 1.39, 95% CI 1.00–1.93, *p* = 0.04) and LFS (HR 1.36, 95% CI 1.00–1.86, *p* = 0.04). Importantly, OS was also influenced by incremental age (HR 1.16, 95% CI 1.00–1.34, *p* = 0.04).

## Discussion

AML incidence increased with age and picked at the sixth–seventh decade of life; it is thus a disease of the elderly, and often at diagnosis, it is highly aggressive in this group of patients. Allo-SCT offers the best curative option with curative potential for these patients, despite the higher risk of complications related to transplant at advanced age as a result of frequent comorbidities and reduced compatible sibling donor availability at this age. Several studies demonstrated the feasibility of allo-SCT with the use of RIC in older population [6–8]. Recently, Rashidi et al. [36] reported 35 and 38% of 3 years LFS and OS, respectively, for elderly AML patients. With the development of new transplant techniques making alternative donors more realistic clinical option, when a MRD donor is not available, the question of which is the best alternative donor and the alternative donor hierarchy is still unanswered. MUD is known to have comparable outcomes to MRD allo-SCT [11–14], but it has the major disadvantage of rather long time for donor identification. On the other hand, Haplo-SCT is increasing worldwide, and it has the main advantage of providing a timely transplant thanks to rapid donor identification for almost all patients in need.

In the present study, we compared the results of allo-SCT from Haplo donors or MUD 10/10 in a large number of AML patients ≥ 60 years, in an attempt to better understand which is the best transplant choice in this setting.

Weisdorf et al. [37], evaluated the role of alternative donors in 740 older AML patients, but only 29 cases of Haplo-SCT were included in the study precluding a comparison for this type of donor.

Chen et al. using ATG based GVHD prophylaxis reported similar outcomes of myeloablative haploidentical SCT in patients aged ≥50 years in comparison to younger adults (24). The same group also confirmed the efficacy of unmanipulated haplo-SCT compared to unrelated donors in young adult with hematological malignancies (25). The feasibility of Haplo-SCT with PT-Cy in the older patients was retrospectively analyzed by Kasamon et al. [26] in 271 patients with hematological malignancies (AML 24%) receiving a RIC regimen with fludarabine, cyclophosphamide, and low dose of TBI. For AML patients aged > 60 years, the 3 years LFS, RI, and OS were 31, 60, and 38%, respectively. These results are in line with ours, especially in terms of OS and LFS, despite the fact that in our analysis, being a registry-based study, we included different conditioning regimes and GVHD prophylaxis. Importantly, given the different type of GVHD prophylaxis in the haplo group, we performed a subgroup analysis according to the use or not of PT-Cy, comparing those two groups vs MUD. Our results confirm the decreased risk of extensive cGVHD for haplo recipients both in the group of PT-Cy and MUD, as well in the group of Haplo with an ATG-based GVHD prophylaxis (without PT-Cy) vs MUD.

In our population, age at transplant was an independent factor associated with reduced OS. Slade et al. [28] recently, retrospectively analyzed the impact of age on Haplo-SCT with PT-Cy and PBSC for AML or myelodysplastic syndrome (MDS), showing a detrimental effect of older age, in accordance with our results.

In a single center study, Blaise et al. [27] recently reported a comparison of outcome of patients older than 55 years receiving Haplo-SCT with PT-Cy, with those receiving MRD or MUD. Despite the low number of patients with AML reported (30%), the results of this study showed that tolerability and efficacy of Haplo-SCT is equivalent to that of MRD and better than MUD. In our population, we did not find difference in OS, LFS, RI, and NRM, between transplantation from Haplo and MUD beside a difference in cGVHD. MUD is associated with higher incidence of cGVHD also when compared with MRD [38]. This could be explained by an effect of minor HLA mismatch and also by the increased use of PBSC in the MUD group, which is an important factor also reported by others [39]. Importantly, the application of PT-Cy as GVHD prophylaxis in the unrelated donor setting is attractive, and some encouraging results are reported [40]. Lastly, Ciurea et al. [29] recently analyzed 43 patients with AML/MDS (median age 61 years) who underwent a Haplo-SCT using PT- Cy. Factors that positively influenced LFS were intermediate/good risk cytogenetics in the first or second remission and younger donors (< 40 years). These results are similar to ours, despite we included different platforms of Haplo-SCT in the current series. Importantly, in our study disease status was one of the main factors associated with outcomes. Using the propensity score analysis, we performed a separate analysis according to disease status, confirming the main results and the reduced risk of cGVHD for patients receiving haploidentical transplant in CR1.

We are aware that our study has some limitations related to its retrospective nature. Importantly, the conditioning regimen and the GVHD prophylaxis are heterogeneous, and thus, we were unable to define a specific drug combination in this particular population of patients.

In order to address the difference in number among the two groups, a propensity score analysis was performed which confirmed the same findings.

## Conclusions

Given our results, we confirm that Haplo-SCT is a valid option in fit AML patients ≥ 60 years, especially in patients in first complete remission, with intermediate/high cytogenetic risk. The rapid availability of Haplo donor and the possible use in further strategies of immunotherapy make this donor source very attractive for high-risk patients. A prospective clinical trial (ClinicalTrials.gov Identifier NCT02623309) comparing Haplo vs MUD in elderly patients without MRD is ongoing to address efficacy, safety, and the evaluation of quality of life. The results of this trial could help in clarifying the role of alternative donors in the elderly, to provide better cure in this high-risk population.

## Abbreviations

a: Acute
allo-SCT: Allogenic stem cell transplantation
ALWP: Acute Leukemia Working Party
AML: Acute myeloid leukemia
ATG: Anti-thymocyte globulins
BM: Bone marrow
Bu: Busulfan
c: Chronic
CI: Cumulative incidence
EBMT: The European Society of Bone Marrow Transplantation
GRFS: Refined graft-versus-host-free, relapse-free survival
GVHD: Graft versus host disease
Haplo-SCT: Haploidentical transplant
KPS: Karnofsky performance status
LFS: Leukemia-free survival
MAC: Myeloablative conditioning
MDS: Myelodysplastic syndrome
MMUD: Mismatched unrelated donors
MRD: Matched related donor
MUD: Matched unrelated donors
NRM: Non-relapse mortality
OS: Overall survival
PBSC: Peripheral blood stem cell
PT-Cy: Post-Tx cyclophosphamide
RI: Relapse incidence
RIC: Reduced intensity conditioning
TBI: Total body irradiation
TCD: T-cell depletion
Tx: Transplant
UCB: Umbilical cord blood
